# Employee resourcing strategies and universities’ corporate image: A survey dataset

**DOI:** 10.1016/j.dib.2018.04.006

**Published:** 2018-04-06

**Authors:** Hezekiah Olubusayo Falola, Olumuyiwa Akinrole Oludayo, Maxwell Ayodele Olokundun, Odunayo Paul Salau, Ayodotun Stephen Ibidunni, Ebe Igbinoba

**Affiliations:** Department of Business Management, Covenant University, Ota, Ogun State, Nigeria

**Keywords:** Manpower planning, Employee attraction, Employee deployment, Employee retention, Corporate image

## Abstract

The data examined the effect of employee resourcing strategies on corporate image. The data were generated from a total of 500 copies of questionnaire administered to the academic staff of the six (6) selected private Universities in Southwest, Nigeria, out of which four hundred and forty-three (443) were retrieved. Stratified and simple random sampling techniques were used to select the respondents for this study. Descriptive and Linear Regression, were used for the presentation of the data. Mean score was used as statistical tool of analysis. Therefore, the data presented in this article is made available to facilitate further and more comprehensive investigation on the subject matter.

**Specifications table**

**Table:** 

Subject area	*Human Resource Management*
More specific subject area	*Employee Resourcing Strategies*
Type of data	*Table, figure*
How data was acquired	*The data were generated through structured questionnaire*
Data format	*Raw, analysed, descriptive and statistical data*
Experimental factors	*Samples consist of Academic Staff of the outstanding six private universities as ranked by different ranking agencies.*
Experimental features	*Employee resourcing strategy is a fundamental factor for building concentration of employees with distinctive capabilities required to promote corporate image.*
Data source location	*Private Universities, Southwest, Nigeria*
Data accessibility	*Data is included in this article*

**Value of the data**

● The data described demographic characteristics of academic staff of the top six ranked private universities in Southwest, Nigeria.
● The study was carried out in order to understand the interplay of respondents’ socio-economic background to issues relating to employee resourcing strategies and university corporate image.
● The data also showed that employee resourcing strategies such as manpower planning, employee attraction, deployment and retention are very helpful in the prediction universities’ corporate image.
● The management of the selected universities can leverage on the data for decision making purposes.
● The outcome is similar to the findings of [2], [6], [7], [8].

## 1 Data

**Fig. 1:**
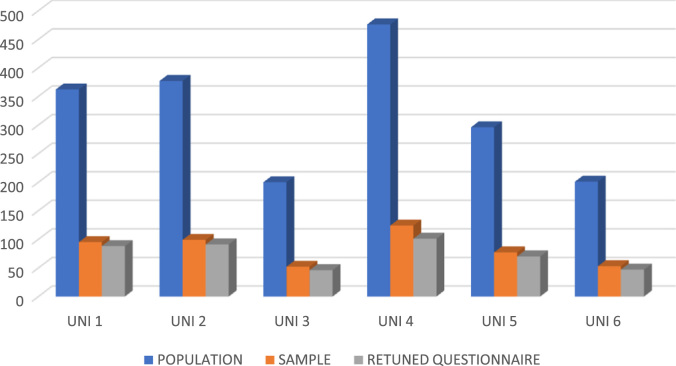
Response rate of administered questionnaire.

The data comprised of demographic characteristics of Academic Staff of selected private universities in Nigeria as well as raw inferential statistical data on the influence of employee resourcing strategies on universities’ corporate image. The response rate of the administered questionnaire as depicted in Fig. 1 shows that out of five hundred copies of questionnaire administered to the Academic Staff of the selected universities only four hundred and forty-four copies were retrieved, which represented 89% response rate.

**Fig. 2:**
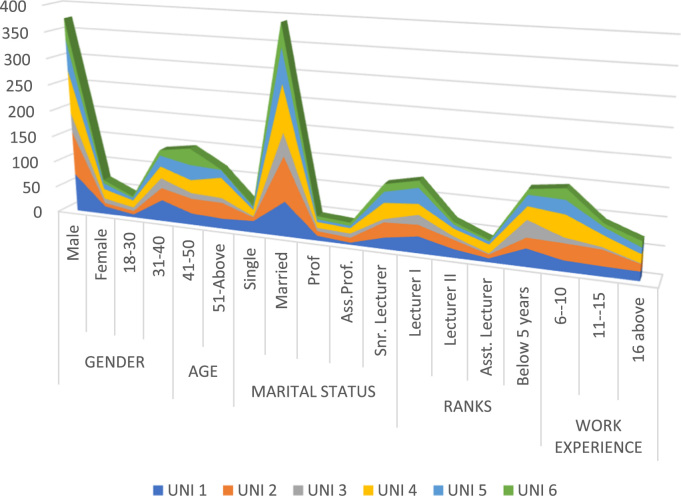
Demographic characteristics of respondents of each university.

The data presented in Table 1 and Fig. 2 show that the Academic Staff of the selected universities were dominated by male representing 85% of the respondents. Similarly, the highest number of respondents were within the age bracket of 31–40. The ranks of the respondents revealed that 22% of the respondents were professors and associate professors, 27% were senior lecturers, 25% were lecturer I while 26% were assistant lecture and graduate assistants.

**Table 1:** Demographic characteristics of academic staff.

**Parameter**	**Characteristics**	**Number**	**Percentage**
**Gender**	Male	375	85
	Female	68	15
		**443**	**100**
			
**Marital status**	Single	151	34
	Married	292	66
		**443**	**100**
			
**Age bracket**	18–30	102	23
	31–40 years	128	29
	41–50 years	109	25
	51years and above	104	23
		**443**	**100**
			
**Work experience**	Less than 5 years	101	23
	6 – 10 years	111	25
	11 – 15 years	122	27
	16 years and above	109	25
		**443**	**100**
			
**Educational qualification**	B.Sc.	33	07
	M.Sc./MBA/	113	26
	Ph.D.	297	67
		**443**	**100**
			
**Ranks**	Prof/Associate Prof.	97	22
	Senior Lecturer	121	27
	Lecturer 1	109	25
	Lecturer II	75	17
	Assistant Lecturer/ Graduate Assistant	41	09
		**443**	**100**

Meanwhile, regression analysis was used to test the effect of the employee resourcing strategies on corporate image. Table 2, Table 3 show the model summary of the analysis, mean scores and standard deviations of measures according to the selected private universities.

**Table 2:** Regression model summary.

Coefficients^a^						
**Model**		Unstandardized coefficients		Standardized coefficients	T	Sig.
		B	Std. Error	Beta		
**1**	(Constant)	1.994	.186		10.713	.000
	Manpower planning strategy	−.168	.057	−.190	−2.920	.004
	Attraction strategy	.253	.027	.377	9.266	.000
	Deployment strategy	.286	.055	.335	5.183	.000
	Retention strategy	.194	.026	.298	7.419	.000
	R	.590^a^				
	R^2^	.348				
	F-value	58.457				

a Dependent variable: Corp_Image.

**Table 3:** Mean scores and standard deviations of measures according to the selected private universities.

	University A		University B		University C		University D		University E		University F	
	Mean	Std. Dev.	Mean	Std. Dev.	Mean	Std. Dev.	Mean	Std. Dev.	Mean	Std. Dev.	Mean	Std. Dev.
**HRPS**	4.2727	.54795	4.4652	.51611	4.4493	.53618	4.3960	.45635	4.6286	.30846	4.3995	.52406
**BI**	4.6553	.39939	4.5348	.42420	4.5145	.41991	4.5578	.49014	4.6381	.40426	4.5734	.45713
**ERS**	3.4034	.74513	3.2692	.86300	3.2500	.75829	3.7252	.63098	3.4929	.67024	3.6321	.82817
**Res I**	4.4053	.57945	3.9231	.72534	3.8188	.70170	4.4290	1.38350	4.4524	.43604	4.2724	.88593
**ESS**	4.7992	2.41300	3.6410	.77177	3.5797	.79329	4.4125	.36560	4.4714	.43773	4.2032	1.27822
**UCR**	4.5909	.39059	4.5879	.48645	4.5109	.48864	4.6881	.41744	4.6500	.30015	4.5711	.48041
**ERetS**	3.8710	.41689	3.9405	.58794	3.2698	.58438	3.2918	.56843	4.0622	1.12178	3.7436	.71221
**UCI**	4.3807	.44201	4.5659	.48425	3.6739	.64306	4.0990	.78428	4.0286	.50994	4.1840	.64915

***HRPS: Human Resource Planning Strategies; BI: Brand Image*****.**

***ERS: Employee Recruitment Strategies***; ***ResI: Research Image***.

***ESS: Employee SelectionStrategies***; ***UCR: University's Corporate Reputation***.

***ERetS: Employee RetentionStrategies***; ***UCI: University's Corporate Identity***.

## 2 Experimental design, materials and methods

In this data presentation, a descriptive design was adopted. The data analysis was done using Statistical Package for Social Sciences (SPSS). The sample for this study consisted of 500 respondents which were randomly drawn from the best six (6) selected private Universities. The motive for the choice of private Universities is because of the level of competition among them when it comes to attracting good local/ international faculty, staff and students. The reason was because very few persons can afford private University education for their children and wards, therefore, attracting good students becomes more competitive. Besides, the choice of Southwest for the study is because it plays host to the twenty-eight (28) private Universities representing 46% out of sixty-one private Universities in Nigeria while the remaining 33 private Universities representing 54% spread across other five (5) geo-political zones in Nigeria. Sequel to the number of private Universities in Southwest Nigeria, the level of competition is extremely high as each University is developing strategies that will give them competitive advantage over others. However, data presented are related to researcher articles [1], [3], [4], [5].

## 3 Ethical considerations

Researchers are mindful of the fact that individuals have a right to be protected from public scrutiny of their private lives. To this end, the researcher ensured that respondents were adequately informed about the objective of this study. In addition, every participant was offered the possibility to stay unknown and their responses treated with utmost confidentiality.
