# Supplementary material for: Employee resourcing strategies and universities’ corporate image: A survey dataset
Source: Data Brief. 2018 Apr 6;18:1378–82. doi: 10.1016/j.dib.2018.04.006 (PMC5996726; doi:10.1016/j.dib.2018.04.006)
Supplement: Supplementary file 1 — Supplementary material [file mmc1.doc]

**Falola Hezekiah Olubusayo (Ph.D)**

Department of Business Management

College of Business and Social Sciences,

Covenant University, Ota, Ogun State, Nigeria

[hezekiah.falola@covenantuniversity.edu.ng](mailto:hezekiah.falola@covenantuniversity.edu.ng)

+234 703 5518 559

**March 22, 2017**

The Editor,

Data In Brief

Dear Sir,

**DECLARATION OF CONFLICT OF INTEREST**

I, Dr. Falola H.O and my colleagues write to declare that there is no conflict of interest traceable to our data paper “**Employee Resourcing Strategies and Universities’ Corporate Image: A Survey Dataset** ”

Thank you.

Yours faithfully,


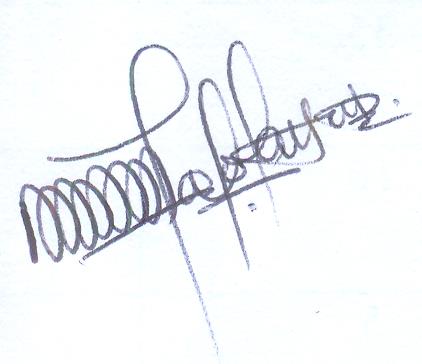


FALOLA H.O (PhD)

**Corresponding Author**
